# Bi-directionalized promoter systems allow methanol-free production of hard-to-express peroxygenases with *Komagataella Phaffii*

**DOI:** 10.1186/s12934-024-02451-9

**Published:** 2024-06-15

**Authors:** Mihail Besleaga, Christian Zimmermann, Katharina Ebner, Robert L. Mach, Astrid R. Mach-Aigner, Martina Geier, Anton Glieder, Oliver Spadiut, Julian Kopp

**Affiliations:** 1 Institute of Chemical, Environmental and Bioscience Engineering, Research Division Integrated Bioprocess Development, Gumpendorfer Straße 1a, Vienna, 1060 Austria; 2 bisy GmbH, Wünschendorf 292, Hofstätten an der Raab, 8200 Austria

**Keywords:** *Komagataella phaffii*, Bi-directionalized promoter, Unspecific peroxygenase, Recombinant protein production, Methanol-free, Derepressed feeding, UPR, ERAD

## Abstract

**Background:**

Heme-incorporating peroxygenases are responsible for electron transport in a multitude of organisms. Yet their application in biocatalysis is hindered due to their challenging recombinant production. Previous studies suggest *Komagataella phaffi* to be a suitable production host for heme-containing enzymes. In addition, co-expression of helper proteins has been shown to aid protein folding in yeast. In order to facilitate recombinant protein expression for an unspecific peroxygenase (*Ano*UPO), we aimed to apply a bi-directionalized expression strategy with *Komagataella phaffii*.

**Results:**

In initial screenings, co-expression of protein disulfide isomerase was found to aid the correct folding of the expressed unspecific peroxygenase in *K. phaffi*. A multitude of different bi-directionalized promoter combinations was screened. The clone with the most promising promoter combination was scaled up to bioreactor cultivations and compared to a mono-directional construct (expressing only the peroxygenase). The strains were screened for the target enzyme productivity in a dynamic matter, investigating both derepression and mixed feeding (methanol-glycerol) for induction. Set-points from bioreactor screenings, resulting in the highest peroxygenase productivity, for derepressed and methanol-based induction were chosen to conduct dedicated peroxygenase production runs and were analyzed with RT-qPCR. Results demonstrated that methanol-free cultivation is superior over mixed feeding in regard to cell-specific enzyme productivity. RT-qPCR analysis confirmed that mixed feeding resulted in high stress for the host cells, impeding high productivity. Moreover, the bi-directionalized construct resulted in a much higher specific enzymatic activity over the mono-directional expression system.

**Conclusions:**

In this study, we demonstrate a methanol-free bioreactor production strategy for an unspecific peroxygenase, yet not shown in literature. Hence, bi-directionalized assisted protein expression in *K. phaffii*, cultivated under derepressed conditions, is indicated to be an effective production strategy for heme-containing oxidoreductases. This very production strategy might be opening up further opportunities for biocatalysis.

**Supplementary Information:**

The online version contains supplementary material available at 10.1186/s12934-024-02451-9.

## Background

Oxidoreductases (EC 1.) belong to an enzyme class present in all domains of life, catalyzing redox reactions essential for a multitude of metabolic reactions [1]. The enzyme chloroperoxidase (EC 1.11.1.10) specifically catalyzes the chlorination of organic compounds using hydrogen peroxide as co-substrate [2]. Chloroperoxidase belongs to the family of heme-containing proteins and was originally isolated from *Aspergillus novoparasiticus* [3]. Even though chloroperoxidase can be used to synthesize fine chemicals and pharmaceutical intermediates, its recombinant production is only scarcely described in the literature. A recombinant production strategy via *Aspergillus niger* was reported; however, the specific activity was only 47*10^− 9^ Units/mol [4]. Trying to produce a non-glycosylated isoform in *Escherichia coli*, Chloroperoxidase resulted in only 2% of the total cellular protein [5].

UPOs (unspecific peroxygenase, EC 1.11.2.1), like Chloroperoxidase, belong to the family of heme thiolate enzymes but possess both, peroxygenase as well as peroxidase activity. UPOs were first discovered in the fungus *Agrocybe aegerita* and since then have been identified in various other fungi [6]. UPOs can be separated into long and short UPOs (depending on their protein size) and are exclusively found in the fungal kingdom, yet their natural function remains unknown [7]. Since UPOs are capable of oxidizing a wide range of organic substrates, including aromatic compounds, alcohols, and aldehydes, they are promising new candidates for applied biocatalysis in the chemical industry [8, 9]. Recently, the toolbox of available UPOs could be broadened tremendously with the identification of a set of 26 novel putative enzyme sequences, whereby half of them could be produced heterologously, and eleven sequences were confidently classified as UPOs [3]. As one of the most promising enzymes of this set, *Ano*UPO was identified as the first-ever expressed UPO from *Aspergillus novoparasiticus*. This enzyme showed a broad substrate acceptance and the potential for reliable production on a microscale as well as higher culture volumes.

Even though the parental organisms of origin are still frequently employed for the production of UPOs, recombinant production has successfully been established for various enzymes of this class [10]. However, protein titers were reported in the lower mg/L range [11]. As heme-containing proteins often possess disulfide bridges, soluble recombinant expression in *E. coli* cytoplasm is impeded [12, 13]. Another opportunity to boost the overall titer of a non-glycosylated isoform is the production in inclusion bodies [14, 15]. This was successfully demonstrated for a hemin containing-protein recently, incorporating the cofactor during the refolding process, yielding an active non-glycosylated enzyme [16]. UPOs have been expressed in *E. coli* previously; however, inclusion body strategies led to low yields requiring soluble protein production in bacterial hosts despite yielding misfolded glycosylation forms [17].

For the production of glycosylated isoforms, however, non-bacterial hosts need to be employed [18]. In this study, we used the ascomycetous yeast *Komagataella phaffii*, previously known as *Pichia pastoris*. *K. phaffii* is used for secreting heterologous proteins at high levels and is capable of producing heme-incorporating enzymes [19, 20]. It is known for its rapid growth rates in defined, low-cost media compared to mammalian cell lines or insect cells [21], even though hypermannosylation is an issue to be considered [22].

High productivity in *K. phaffii* is often the result of methanol-based induction mechanisms [23]. To this end, the alcohol-oxidase promoter (P_*AOX1*_) is frequently employed [24]. As an alternative to methanol-based induction, constitutive promoters can be used for recombinant protein formation [25].

For constitutive recombinant protein expression in *K. phaffii*, the promoters P_*GAP*_, P_*UPP*_, and P_*HHT1*_ are conventional solutions, all originating from *K. phaffii* itself [26, 27]. While P_*GAP*_ and P_*UPP*_ (commercially available variant of the *K. phaffii* P_*GCW14*_) are referred to as strong constitutive regulatory elements, P_*HHT1*_ is considered a medium to intermediate constitutive strength promoter. Importantly, constitutive recombinant protein production does not allow for a separation of the process in dedicated growth- and production-phases, resulting in decreasing biomass yields and, thus, lower volumetric target protein expression [28–30]. To avoid the inability of switching the induction mechanism on and off, derepressive activated promoters are a sustainable alternative to methanol-based induction. By a simple reduction of the feeding rate, the promoter can be activated without any additional change in carbon source [31].

The P_*DF*_ promoter, originating from *Hansenula polymorpha*, belongs to the group of derepressive promoters, with an additional activity boost upon methanol addition [32]. The P_*DC19*_ promoter is a slightly modified version of the P_*CTA1*_ (commercially available as P_*DC*_) promoter originating from *K. phaffii*, with similar induction behavior as the P_*DF*_ promoter [33]. Both promoters can be induced under derepressive feeding conditions or via methanol addition.

In previous studies, methanol-based induction was suggested to be advantageous for the successful recombinant production of heme-containing enzymes in *K. phaffi* [33, 34]. Still, methanol is a toxic reductant with a high heat of combustion, causing high oxygen consumption [35] and being lethal to *K. phaffii* cells at concentrations between 2 and 5% v/v [36]. Hence, implementation, of methanol-based induction mechanisms on an industrial scale demands complex infrastructure, such as an explosion-safe environment and strong cooling facilities to reduce the heat caused by methanol consumption [30]. Even though the cooling issue can be circumvented by utilizing a Mut^S^ strain, methanol-based induction triggers the misincorporation of O-methyl-L-homoserine instead of methionine in Mut^S^ strains, demanding different induction mechanisms [37–39]. Moreover, methanol degradation has been reported to cause oxidative stress and enhance the unfolded protein response (= UPR) pathway [40]. Protein secretion and protein degradation pathways are affected as a consequence, causing shifts in UPR and the endoplasmic reticulum-associated degradation (ERAD) in *K. phaffi* [40]. To conclude, there is a dire need for well-established methanol-free promoter systems, easing industrial recombinant enzyme production with *K. phaffi*.

An intriguing alternative to methanol-inducible systems, which allow the possibility of achieving similar yields in enzymatic activity, are bi-directionalized methanol-free promoter systems. There, a chaperone or any other helper protein aiding the target protein folding mechanism is co-expressed [26], potentially boosting enzymatic activity [41].

In this study, we investigated the recombinant expression of a UPO with *K. phaffi* utilizing a synthetic bi-directionalized promoter-based system. A mock strain, producing the target enzyme without additional protein disulfide isomerase (= PDI) expression in a mono-directional manner, was compared to strains based on synthetic bi-directionalized constructs with targeted PDI co-expression. Initially, different promoter combinations for PDI expression were screened for, and the best-producing clone was chosen for bioreactor cultivation. The chosen promoter for target enzyme expression (P_*DF*_) can be activated by derepressed feeding and is referred to an additional activity boost by methanol addition. Hence, both induction strategies were initially investigated for the resulting enzyme productivity. An RT-qPCR analysis was performed to decipher differences in productivity. We observed altered protein secretion pathways (UPR and ERAD) depending on the induction mechanism and the respective strain.

## Methods

### Strain engineering

All synthentic bi-directionalized expression constructs were designed and constructed based on the bisy proprietary standard vector pBSY5Z, employing the strong derepressive/methanol inducible P_*DF*_ for expression of the *Ano*UPO [3]. The *Ano*UPO coding sequence, including the native signal sequences (KAB8223135.1) and the PDI gene of *K. phaffii*, were codon optimized for expression in *K. phaffii* using the high methanol codon usage table, as described by Abad et al. and ordered as synthetic DNA [42]. *Ano*UPO originates from the parent organism *Aspergillus novoparasiticus*, which is 31 kDa in size, has a pI of 5.56, and carries a heme-cofactor. It has five potential N-glycosylation sites and one cysteine residue for a intermolecular disulfide bridge, making the recombinant production of this enzyme challenging.

**Fig. 1:**
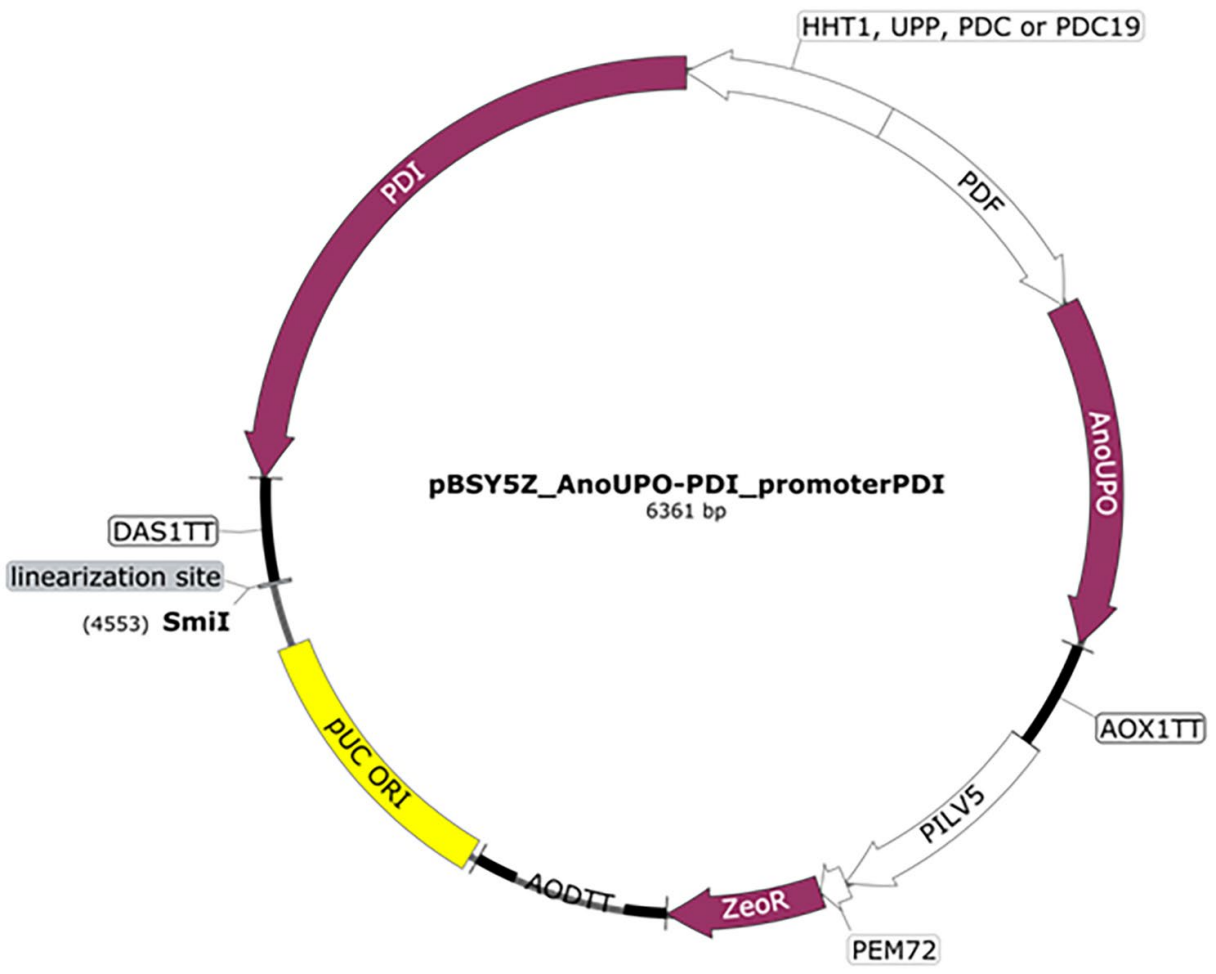
Representative plasmid map of bi-directionalized expression constructs pBSY5Z_*Ano*UPO-PDI_promoterPDI. Vectors contain the coding sequence for the *Ano*UPO (KAB8223135.1) with its native signal sequence and the P_*DF*_ as promoter controlling its expression, the PDI with different promoter sequences controlling its expression, parts necessary for bacterial propagation (pUC ori), selection in bacteria and yeast (Zeocin resistance cassette: P_ILV5-P_EM72-ZeoR-AOD_TT) and a unique restriction site for linearization of the vector prior to transformation (SmiI)

Promoter sequences were either amplified from the genome of *K. phaffii* BSYBG11 (*HHT1*) or from bisy proprietary standard vectors (P_*UPP*_, P_*DC*_, P_*DC19*_). Cloning of all four bi-directionalized expression constructs was done using isothermal assembly adapted from Gibson et al. using PCR amplified DNA parts containing terminal sequence homologies to the respective adjacent vector parts [43]. In total, four expression constructs were produced: pBSY5Z_*Ano*UPO-PDI_P_*HHT1*_, pBSY5Z_*Ano*UPO-PDI_P_*UPP*_, pBSY5Z_*Ano*UPO-PDI_P_*DC*_, pBSY5Z_*Ano*UPO-PDI_P_*DC19*_ (Fig. 1). After sequence verification by Sanger sequencing constructs were linearized with *Sm*iI, and 1 µg of DNA was used for the transformation of electrocompetent *K. phaffii* BSYBG11 cells following Lin-Cereghino et al. [44]. For each construct,>80 clones were cultivated in a microscale, as described previously [45]. Briefly, cells were cultivated in minimal buffered media (pH 6) with 1% (w/v) glucose as the sole carbon source (BMD1) for 60 h. The batch phase was followed by a methanol induction phase for a further 60 h, where the methanol concentration was kept at 0.5% (v/v). After 120 h, the culture supernatant was harvested by centrifugation and analyzed for enzymatic activity using ABTS as a substrate, as described previously [3]. The re-screening was done following the same cultivation protocol using single colonies of selected clones (gained by a dilution streak out), which were cultivated as biological triplicates. The rescreening was always performed with the top three producers of each construct (in regard to enzymatic activity) as well as utilizing two clones resulting in intermediate activity.

### Bioreactor cultivations

All preculture cultivations were carried out using a yeast nitrogen base media (YNBM). Preculture media consisted of 0.1 M potassium phosphate buffer pH 6; yeast nitrogen base w/o Amino acids, 13.4 g/L; (NH_4_)_2_SO_4_, 5 g/L; biotin, 400 mg/L; glycerol, 20 g/L. Batch media was composed of basal salt medium (BSM) consisting of 85% (v/v) phosphoric acid, 26.7 mL/L; CaSO_4_*_2_H_2_O, 1.17 g/L; K_2_SO_4_, 18.2 g/L; MgSO_4_*7H_2_O, 14.9 g/L; KOH, 4.13 g/L; glycerol, 20 g/L supplied with trace elements. The feed solution was composed of either 400 g/L glycerol or a mixed feed solution of 400 g/L glycerol and 40 g/L methanol, depending on the respective process conditions.

Preculture media was inoculated with 1.5 mL of cryo stock solution, with cryo stocks being stored at -80 °C and subsequently cultivated for 20 h at 30 °C, 230 rpm in a shaking incubator (Multitron, Infors HT, Basel, Switzerland).

The cultivations were performed in a Minifors 2 bioreactor system (max. working volume: 2 L; Infors HT, Bottmingen, Switzerland). The cultivation offgas flow was analyzed online using offgas sensors - IR for CO_2_ and ZrO_2_ based for O_2_ (Blue Sens Gas analytics, Herten, Germany). Process control and feeding were performed using EVE software (Infors HT, Bottmingen, Switzerland). During cultivation, the temperature was maintained at 30 °C, pH was kept constant at 5 and controlled with base addition only (12.5% NH_4_OH), while acid (10% H_3_PO_4_) was added manually, if necessary. The pH was monitored using a pH-sensor EasyFerm Plus (Hamilton, Reno, NV, USA). Aeration was carried out using a mixture of pressurized air and pure oxygen at two vvm. Oxygen was added accordingly to keep dissolved oxygen (dO_2_) always higher than 30%. The dissolved oxygen was monitored using a fluorescence dissolved oxygen electrode Visiferm DO (Hamilton, Reno, NV, USA).

### Cultivation scheme and q_s_ adaption

Inoculation was performed with 1/10 of the batch media volume. Preculture showed an OD_600_ of approximately 10 after cultivation for approximately 20 h. The batch process, performed at 30 °C, took around 12 h and was finished, visible by a drop in the CO_2_-signal. The 20 g/L of glycerol usually resulted in a biomass of 9–10 g/L. After the batch was finished, a feed was started using varying conditions.

Static feed-forward q_s_-controls were performed [46, 47], where exponential feeding profiles were established according to Eq. 1 to keep q_s, C_ constant [46–49]:

1 $$ F\left(t\right)=\frac{{q}_{s, C}\text{*}X\left(t\right)\text{*}{\rho }_{f}}{{c}_{f}}$$

With F being the feed rate [g/h], q_*s, C*_ the specific glycerol uptake rate [g/g/h], X(t) the absolute biomass [g], ρ_F_ the feed density [g/L], and c_F_ the feed concentration [g/L] respectively. For applied control strategies, adaption of the q_s, C_ during the induction time is performed based on Eq. 1.

### Process analytics

Samples were always taken after inoculation, upon the end of the batch phase, and bi-daily during the fed-batch until the process was finished. Biomass was measured using OD_600_ and DCW (dry cell weight). Optical density (OD_600_) was measured using an ONDA V-10 Plus spectrophotometer (GIORGIO BORMAC, Italy). Since the linear range of the used photometer is between 0.1 and 0.8, samples were diluted with dH_2_O to stay within that range. The dry cell weight was determined by vortexing the sample, pipetting 1 mL of the sample solution in a pre-tared 2 mL Eppendorf-Safe-Lock Tube (Eppendorf, Hamburg, Germany), and centrifuging it for 10 min at 9000 g at 4 °C. After centrifugation, the supernatant was used immediately for at-line HPLC measurement (see beneath), while the pellet was re-suspended with 1 mL of 0.9% (w/v) NaCl solution and centrifuged at the same conditions. Afterward, the pellet was dried for at least 72 h at 105 °C.

Glycerol and methanol concentrations were measured via an anion exchange HPLC (Thermo Scientific, Waltham, MA, USA). The eluent was 0.1% H_3_PO_4_ at a flow rate of 0.5 mL/min using an Aminex HPLC column (HPX-87 H Column, 300*7.8 mm; Biorad, Hercules; CA, USA). Using this method, glycerol accumulation could be detected. Prepared standards had concentrations covering the range from 1 to 50 g/L of glycerol. Chromatograms were analyzed using Chromeleon Software (Dionex, Sunnyvale, CA, USA).

### Product analytics

For identification of total protein concentration in the supernatant, 1 mL sample (Eppendorf, Hamburg, Germany) was centrifuged for 10 min at 9000 g at 4 °C. After the centrifugation step, the supernatant was collected and analyzed according to the Bradford protocol [50], while the pellet was discarded. The reaction mixture consisted of 200 µL of Bradford reagent solution mixed with 5 µL of a supernatant sample. The change in absorbance at 595 nm was monitored using a Tecan Infinite M200 PRO (Tecan, Männedorf, Switzerland) after 10 min of incubation.

Enzyme activity was measured using the same platereader in a 96-well plate. The reaction mixture, per well, consisted of 170 µL of ABTS solution (5 mM ABTS in 50 mM KH_2_PO_4_, pH 5), 10 µL of a sample (diluted, if required), and 20 µL of H_2_O_2_ (final concentration one mM). After the addition of H_2_O_2_, the plate was immediately placed in the platereader at 30 °C, and the change of absorption at 420 nm was monitored for 2 min. The volumetric enzyme activity was calculated according to Humer et al. 2020 [16].

### Shake flask cultivations to determine RT-qPCR reproducibility

All shake flask cultivations were carried out with the *K. phaffi* BSYBG11-based strains carrying the abbreviations *Ano*UPO and *Ano*UPO-PDI. Preculture media from bioreactor cultivations was used as culture media for shake flask cultivations. Cultivations in the shake flasks were performed in triplicates to investigate the effects of starvation, i.e., derepression, methanol (1% v/v), and glycerol addition (20 g/L). Separate Ultra Yield Flasks were used for overnight culture of *Ano*UPO and *Ano*UPO-PDI producing strains in an Infors shaking incubator (Multitron, Infors HT, Basel, Switzerland) at 30 °C and 230 rpm. The overnight cultures were used to inoculate (inoculum size represented 10% of end volume) new shake flasks with the preculture media containing methanol (1% v/v pulse), glycerol (end concentration 20 g/L) or no C- source (starvation) in triplicates. Biomass samples were withdrawn after 8 h for RT-qPCR analysis.

### RT-qPCR analytics

Approx. 0.1 g of yeast cells were re-suspended in 1 ml RNAzol RT (Sigma-Aldrich) and lysed using a Fast-Prep-24 (MP Biomedicals, Santa Ana, CA, USA) with 0.5 g of glass beads (1 mm diameter) twice at six m/s for 30 s. Samples were incubated at room temperature for 5 min and then centrifuged at 12,000 g for 5 min. 650 µl of the supernatant sample were mixed with the same amount of ethanol. RNA was then isolated using the Direct-zol RNA Miniprep Kit (Zymo Research, Irvine, CA, USA) according to the manufacturer’s instructions. This Kit includes a DNAse treatment step. The concentration and purity were measured using the NanoDrop ONE (Thermo Fisher Scientific, Waltham, MA, USA).

500 ng of isolated total RNA was reverse transcribed using the LunaScript RT SuperMix (NEB) according to the manufacturer’s instructions. The resulting cDNA was diluted 1:50, and 2 µl was used as a template in a 15 µl reaction using the Luna Universal qPCR Master Mix (NEB) according to the manufacturer’s instructions. The primers used are listed in additional data Sect. 3. All reactions were performed in technical duplicates on a Rotor-Gene Q system (Qiagen, Hilden, Germany). Calculations of the relative transcript levels were performed according to the Pfaffl method [51] using the reference genes RSC1 and TAF10 for normalization according to [52].

## Results and discussion

In this study, we aimed to avoid the use of the inducer methanol due to the aforementioned reasons whilst achieving high yields of hard-to express peroxygenases using *K. phaffii*. Hence, we (i) compared the recombinant expression of *Ano*UPO from a conventional mono-directional expression system to a bi-directionalized system co-expressing protein disulfide isomerase (PDI) as a folding helper and (ii) investigated whether a derepressed or a methanol-induced feeding strategy yields higher volumetric and specific enzyme productivities.

### Strain screening

Looking into literature, for many challenging target proteins chaperone or foldase co-expression has shown a strong positive effect on recombinant protein titer or quality [53], especially the eukaryotic chaperon protein disulfide isomerase (PDI) is oftentimes cited as a highly successful “expression partner” [54]. Since the benefits of PDI co-expression are typically related to disulfid bond formation and many UPOs form intra- or intermolecular disulfid bridges it has previously been suggested to use PDI to increase the titer of recombinantly produced UPOs [55, 56, 57, 58]. Following up on this theory, we tested the effects of PDI co-expression on the production of the unspecific peroxygenase *Ano*UPO, a short-type UPO with one cysteine residue potentially enabling dimerization via disufid bond formation. To facilitate cloning and library-style shuffling of promoter sequences we used a bi-directionalized promoter system, as described by Vogl et al. [26], for *Ano*UPO and PDI co-expression and compared the resulting enzymtic activity to standard monodirectional *Ano*UPO expression. In the monodirectional and bi-directionalized system, the expression of *Ano*UPO was controlled by P_*DF*_ [33]. In the bi-directionalized constructs, the PDI was co-expressed using different promoter systems with the aim of balancing UPO and PDI expression to eventually enhance enzyme productivity and, by association, potentially also activity and/or quality.

To identify a suitable co-expression system, we screened four different bi-directionalized promoter combinations in regard to their *Ano*UPO productivity. These systems contained differently regulated promoters, comparing constitutive with derepressed and methanol-inducible expression systems of the PDI. For each regulatory mechanism, a strong and a medium strength promoter were tested. For constitutive expression, the P_*HHT1*_ (promoter of *K. phaffii* histone gene HHT1) and the P_*UPP*_ (a commercially available variant of the *K. phaffii* P_*GCW14*_) were chosen as medium and strong promoter sequence, respectively. For derepressed and inducible expression, the P_*DC*_ (a commercially available variant of the *K. phaffii* P_*CTA1*_) and an in-house created, stronger variant thereof (P_*DC19*_, non-published data) were evaluated [33].

For each construct, the mean activity of more than 80 clones was compared to an averagely performing *K. phaffi* strain expressing the *Ano*UPO from a mono-directional construct. After the initial screening, five clones per construct were chosen as candidate strains for upscaling, and their activity was re-evaluated in biological replicates (Table 1).

**Table 1:** Strain screening of different bi-directionalized promoter constructs monitored for target enzyme expression (*Ano*UPO). The expression of *Ano*UPO was always regulated by the P_*DF*_ promoter. The control strain was an averagely performing *K. phaffi* strain expressing the *Ano*UPO from a mono-directional construct, which is later abbreviated as the *Ano*UPO strain. The additional co-expression of the protein disulfide isomerase (PDI) was varied under two inducible, P_*DC*_ and P_*DC19*_, and two constitutive, P_*UPP*_ and P_*HHT1*_, promoters. Either four or five of the most promising bi-directionalized clones were analyzed for their *Ano*UPO activity (using ABTS as a substrate) in triplicates

	Activity [U/mL] ± relative standard deviation [%]
averagely performing mono-directional reference = *Ano*UPO strain	0,0101 ± 9,90%
PDC clone 1	0.0127 ± 3.94%
PDC clone 2	0.012 ± 5.83%
PDC clone 3	0.0118 ± 0.85%
PDC clone 4	0.0131 ± 8.40%
PDC clone 5	0.0129 ± 6.98%
PDC 19 clone 1 (= *Ano*UPO-PDI strain)	0.0153 ± 7.19%
PDC 19 clone 2	0.0139 ± 5.04%
PDC 19 clone 3	0.0139 ± 7.91%
PDC 19 clone 4	0.013 ± 9.23%
PDC 19 clone 5	0.0124 ± 8.06%
UPP clone 1	0.0089 ± 6.74%
UPP clone 2	0.0108 ± 9.26%
UPP clone 3	0.094 ± 7.45%
UPP clone 4	0.0105 ± 4.76%
HHT1 clone 1	0.0111 ± 9.01%
HHT1 clone 2	0.0112 ± 8.93%
HHT1 clone 3	0.0121 ± 9.92%
HHT1 clone 4	0.012 ± 7.50%
HHT1 clone 5	0.0117 ± 12.82%

Results indicated that for three out of four bi-directionalized constructs, the additional PDI expression positively influenced enzymatic activity in the supernatant. For the constitutive mode of chaperone expression, low-level PDI production (P_*HHT1*_) resulted in higher enzyme activity compared to high-level constitutive expression. Interestingly, strong constitutive expression of PDI (via P_*UPP*_) seemingly even had a slightly negative effect on enzyme production. A superior mode of action was found for the simultaneous production of *Ano*UPO and PDI using derepressed/methanol-induced promoters for PDI expression (P_*DC*_ and P_*DC19*_). The highest enzymatic activity in the supernatant was obtained for the P_*DC19*_-P_*DF*_ promoter combination, which corresponds with literature, where high yields of recombinant protein for methanol-inducible promoters are described [59]. Therefore, the clone co-expressing the PDI under the control of the P_*DC19*_, showing a 1.4-fold increased activity (*Ano*UPO-PDI strain) compared to the mono-directional reference strain (*Ano*UPO strain), was chosen for further work.

### Physiological characterization

Controlled fermentations of both clones were performed in a 2 L scale under different process conditions to physiologically characterize the two yeast strains.

The first bioreactor runs allowed to determine the specific substrate uptake rates (q_s max glycerol_ in g/g/h) and maximum growth rates (µ_max_ in h^-1^), respectively, under methanol-free conditions (Table 2).

**Table 2:** Maximum specific growth rates and specific substrate uptake rates of the two used *K. phaffi* strains, *Ano*UPO and *Ano*UPO-PDI

	*Ano*UPO	*Ano*UPO-PDI
µ_max_ glycerol [h^-1^]	0.142 ± 0.004	0.161 ± 0.007
q_s max glycerol_ [g/g/h]	0.284 ± 0.008	0.322 ± 0.014

**Fig. 2:**
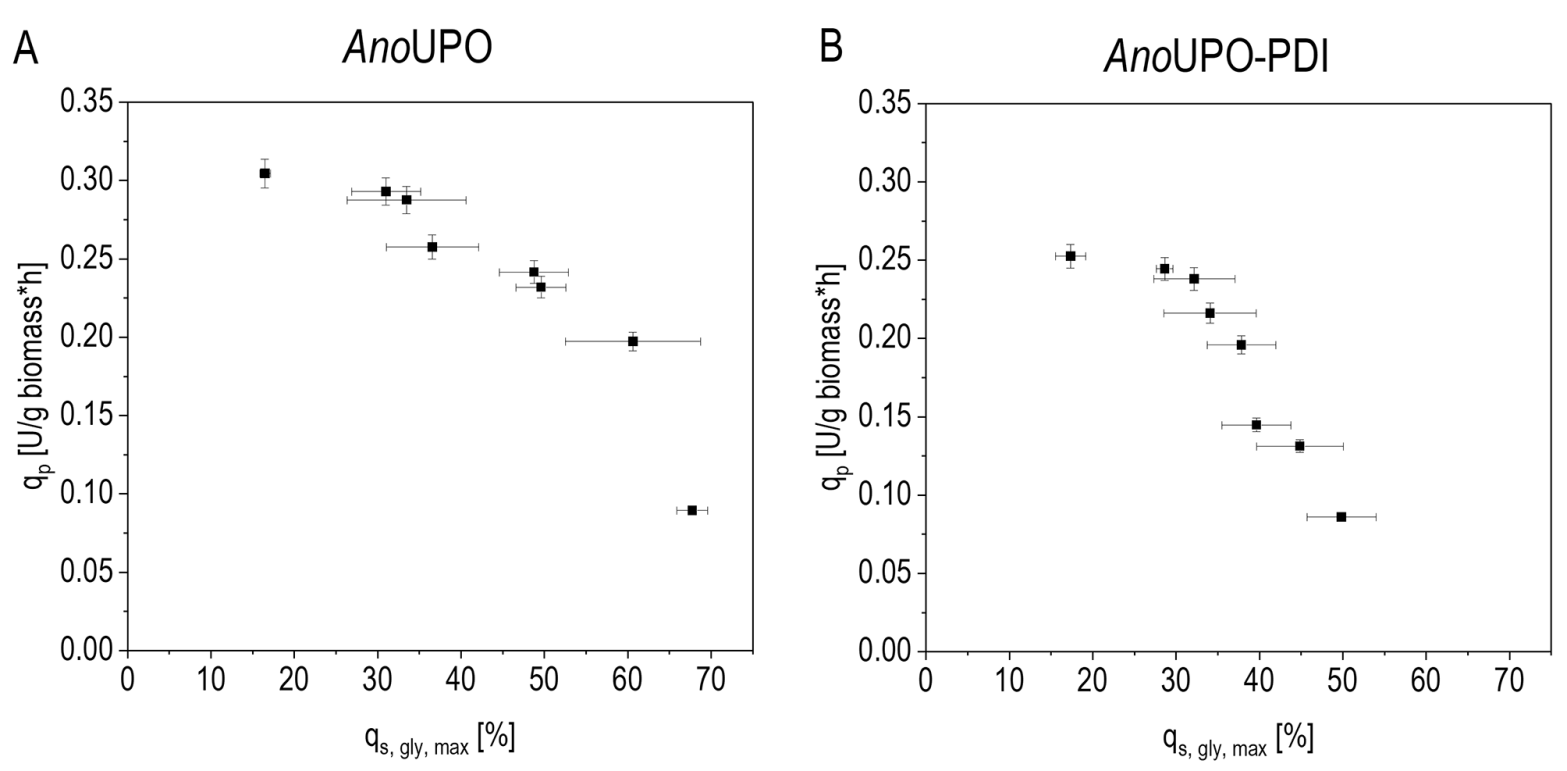
The mechanistic dependence of q_p_ and q_s glycerol_ is shown. Controlled fed-batch cultivations at different specific glycerol uptake rates q_s glycerol_ were performed; the specific productivity for *Ano*UPO (q_p_) was investigated as a function of q_s glycerol_ for both recombinant yeast strains (**A** and **B**, respectively)

In a series of controlled fed-batch experiments, both strains were examined for physiology and recombinant protein expression as a function of the specific glycerol uptake rate q_s glycerol_ (Fig. 2).

For both strains, full derepression was achieved at 0 to 30% of q_s glycerol_ (Fig. 2), indicating the derepressive induction mode referred to in literature [31]. Tendencies indicated that specific productivity should increase at lower feeding rates (Fig. 2), which is in accordance with the literature on derepressive systems [60]. The strains behaved very similarly in this respect, i.e., the physiology does not seem to be affected by the co-expression of the chaperone PDI. However, the cell-specific productivity (q_p_) of the *Ano*UPO-PDI strain was found to be surprisingly slightly lower than that of the *Ano*UPO strain.

Based on the results of the screening, methanol uptake rates were investigated at derepresssible glycerol-feeding rates (q_s glycerol_ =30%). This was done, as literature suggests a boost in P_*DF*_ and P_*DC*_ promoter activity, when additionally supplementing methanol to derepressible conditions [33, 34].

To determine feeding ratios for bioreactor cultivations, the feeding rate at 30% of µ_max_ was investigated for its co-methanol consumption to determine whether methanol addition boosts activity. The screening was done using previously published screening methods for mixed-feed methanol uptake with *K. phaffii* [61, 62]. Thereby, q_s max MeOH_ is a crucial parameter in feeding strategies with *K. phaffii*, as overfeeding beyond 2% v/v methanol in the supernatant triggers cell lysis [37, 38]. Table 3 lists the maximum specific methanol uptake rates q_s max methanol_ at specific q_s glycerol_.

**Table 3:** Maximum specific uptake rates of methanol (q_s max MeOH_ ) at 30% of q_s max glycerol_

*Ano*UPO		*Ano*UPO-PDI	
q_s, glycerol_ [%]	q_s max MeOH_ [mg/g/h]	q_s, glycerol_ [%]	q_s max MeOH_ [mg/g/h]
31.2	20.7 ± 2.6	30.7	19.6 ± 2.5

Results from Table 3 indicate that at 30% of q_s, glycerol, max_ (the specific glycerol uptake rate that allows complete derepression, as determined before) around 20 mg of methanol per gram of biomass can be taken up per hour.

As a next step, the effect of methanol and derepressive induction on specific *Ano*UPO product formation was assessed. To avoid potential methanol overfeeding of the cultivations due to a decreasing biomass yield over time, the specific uptake rate of methanol was set to 50% of the maximum possible methanol uptake rate. This strategy was also successfully reported for the production of the akin heme-containing enzyme horseradish peroxidase with *K. phaffi* [63]. Hence, cultivations were operated at different specific uptake rates of glycerol (q_s glycerol_) with co-feeding of methanol at a ratio of 50% of q_s max MeOH_. Experiments were performed in a dynamic manner for this initial screening.

**Table 4:** Specific *Ano*UPO productivities and product purities in the culture supernatant in different derepressed and glycerol-methanol mixed-feed fermentations of the two strains *Ano*UPO and *Ano*UPO-PDI. The factor was calculated from the specific activity (either [U/g/h] or spec. activity [U/mg protein]) of the derepressed experiments divided by the mixed feed strategy at the respective comparable feeding rate. For each strain, four set-points in total were screened for, resulting in two set-points to evaluate mixed feeding and two set-points to test for the effects of derepresible feeding

	*Ano*UPO			*Ano*UPO-PDI		
	q_s, glycerol_ [%]	q_p_ [U/g/h]	spec. activity [U/mg protein]	q_s, glycerol_ [%]	q_p_ [U/g/h]	spec. activity [U/mg protein]
Derepressive	31.7	0.291	1.56	28.6	0.244	1.80
Mixed feed	27.3	0.184	1.25	26.3	0.165	1.43
Factor		1.58	1.25		1.48	1.26
Derepressive	16.5	0.305	2.23	17.3	0.253	2.77
Mixed feed	19.2	0.131	0.686	14.9	0.121	0.753
Factor		2.33	3.25		2.09	3.68

When comparing cell-specific productivity and protein-specific activity of *Ano*UPO (Table 4), we observed two effects: First of all, higher amounts of active *Ano*UPO could be produced with derepressive feeding strategies when compared to the mixed feed strategies. This is contrary to literature since previous reports recorded higher production titers for heme-incorporating peroxidases upon methanol-based feeding strategies [3, 20, 33]. The second result obtained from the screening was that co-expression of the enzyme PDI reduces the total q_p_ of the target enzyme *Ano*UPO; however, it is produced at a higher specific activity. The reduced overall productivity could be potentially explained by the co-expression of PDI, requiring a certain amount of intracellular energy (ATP) and material in the form of amino acids. Consequently, less energy would be available for the production of the UPO.

Methanol metabolization triggers the expression of alcohol oxidases, catalases, and a variety of other enzymes of the methanol metabolic pathway [64]. Hence, it is not surprising that the protein-specific *Ano*UPO activity [U/mg protein] seems to decline when methanol is used for induction. Even though the *Ano*UPO-PDI strain showed a slightly reduced specific productivity (q_p_) compared to the *Ano*UPO strain, a slight increase in product quality could be achieved. Since the presence of PDI facilitates the formation of disulfide bridges, the PDI co-expression presumably reduces the export of misfolded target UPO.

To conclude, additional methanol addition did not aid recombinant enzyme expression even though reported in literature [33]. PDI co-expression resulted in lower productivity in this experimental setup, however, in slightly enhanced specific activities.

### Establishing a production process for *Ano*UPO

In order to determine a suitable process for the production of *Ano*UPO, the best conditions were determined using the dynamic screening approaches. The goal, thereby, was to establish a process for mixed feeding and a process for a derepressive induction strategy, both of which were executed in static experiments. Thereby, induction at the desired set-point (constant q_s_) for 72 h was done to monitor time-dependent target enzyme secretion, as described for other peroxygenases [65]. Applied conditions are as follows, “Mixed feed cultivation”: q_s, glycerol, max_ at 30% = adjusted q_s, glycerol_ of 75 mg/g/h with q_s, methanol_ at 20 mg/g/h and “Derepressive cultivation”: q_s, glycerol, max_ at 20% = adjusted q_s, glycerol_ of 50 mg/g/h.

**Fig. 3:**
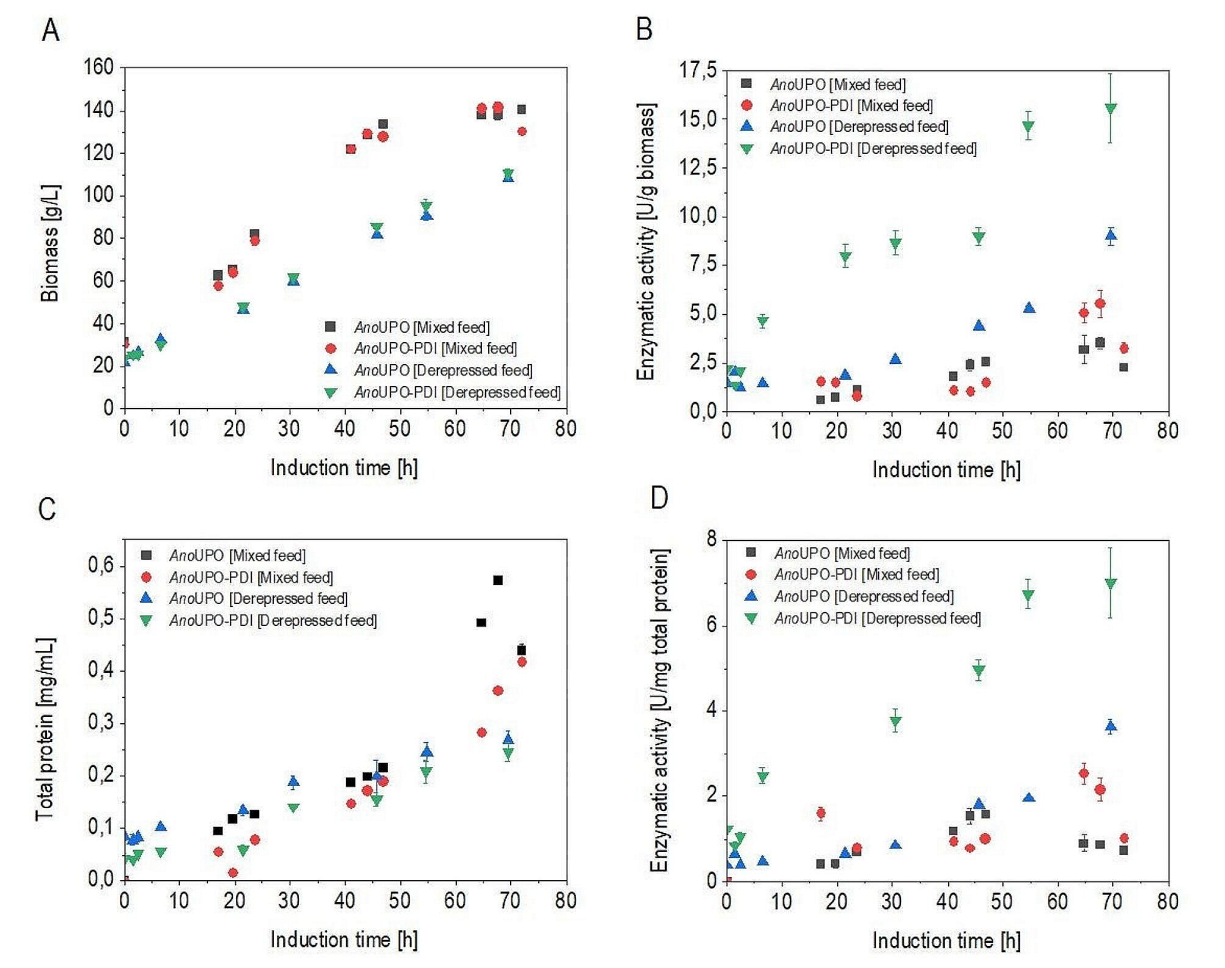
Cultivations performed at conditions resulting in highest q_p_ at respective conditions: “Mixed feed cultivation” with 30% of q_s, glycerol, max_ = adjusted q_s, glycerol_ of 75 mg/g/h with q_s, methanol_ at 20 mg/g/h and “Derepressive cultivation” with q_s, glycerol, max_ at 20% = adjusted q_s, glycerol_ of 50 mg/g/h **A**) Biomass concentration for the *Ano*UPO and *Ano*UPO-PDI strains are shown for derepressed and mixed feed cultivation over time of induction; **B**) indicating the biomass-specific expression of the enzyme *Ano*UPO for the respective cultivation of the two strains; **C**) The total protein concentrations of the respective supernatants for the *Ano*UPO and *Ano*UPO-PDI strains in the respective cultivation conditions; **D**) the protein-specific expression (i.e., purity) of the enzyme *Ano*UPO for the respective cultivation of the two strains

While biomass production in the mixed-feed cultivation stagnates towards the end of the cultivation time (Fig. 2A.), biomass growth in the derepressive cultivation was not affected negatively. Even though the set µ was slightly higher in the mixed feed cultivation compared to the derepressive cultivation, total biomass is far from being physiologically limited in *K. phaffii* cultivations [66]. Hence, we assume the declining biomass indicates cell stress during mixed feeding. When maintaining ideal production set-points, it was also observed that the *Ano*UPO-PDI strain was producing higher levels of active enzyme (Fig. 3B). As discussed previously, the PDI is likely to assist the folding of the active UPO, thereby achieving higher productivity. We attribute this effect not being so strongly pronounced in screening experiments due to alternating feeding rates during screening, causing dynamic process conditions. As promoters for *Ano*UPO and PDI expression are not completely alike, dynamic feeding conditions might have been causing a potential intracellular misbalancing of ATP between UPO and PDI production. Notably, the *Ano*UPO expression in methanol-fed cultivations collapses towards the end of cultivations, which could only, to a certain extent, be salvaged by PDI co-expression. No decrease in productivity is observed in the derepressive induced cultivations (Fig. 3B).

Figure 3C indicates that methanol-based induction strategies yielded a higher amount of total protein in the supernatant when compared to the derepressed feeding strategies. This is consistent with the literature, where P_*DF*_ is described to be strongly induced with methanol compared to methanol-free derepression [33]. In the derepressive cultivation, total protein formation was comparable between the *Ano*UPO and the *Ano*UPO-PDI strain, whereas, in the mixed-fed cultivation, the *Ano*UPO strain yielded slightly higher protein levels (0.1 g/L difference). Still, when looking at the protein-specific activity (Fig. 3D), the benefit of the PDI co-expression could clearly be observed: comparing *Ano*UPO activity in between respective cultivations, the *Ano*UPO-PDI shows a higher enzymatic activity compared to the *Ano*UPO reference strain. Additionally, as Fig. 3D indicates, the specific *Ano*UPO production was superior in derepressive cultivations over mixed-fed cultivations.

In summary, the derepressive *Ano*UPO-PDI cultivation yielded in the highest specific and total *Ano*UPO production (~ 7 U/mg total protein, Fig. 3D), resulting in a 93% yield increase compared to the *Ano*UPO cultivation conducted at the same conditions.

### RT-qPCR analysis of production runs

In order to monitor the effects of the executed cultivations on a molecular level, RT-qPCR analyses of selected targets, amongst them the recombinant *Ano*UPO and PDI genes as well as *K. pfhaffii* endogenous genes, were performed as described recently [52].

Both the P_*DF*_ and the P_*DC19*_ promoter, regulating the expression of the *Ano*UPO and the PDI, respectively, showed high transcript formation once the induction phase was started, with transcription levels independent from the cultivation strategy (additional data Sect. 1).

**Fig. 4:**
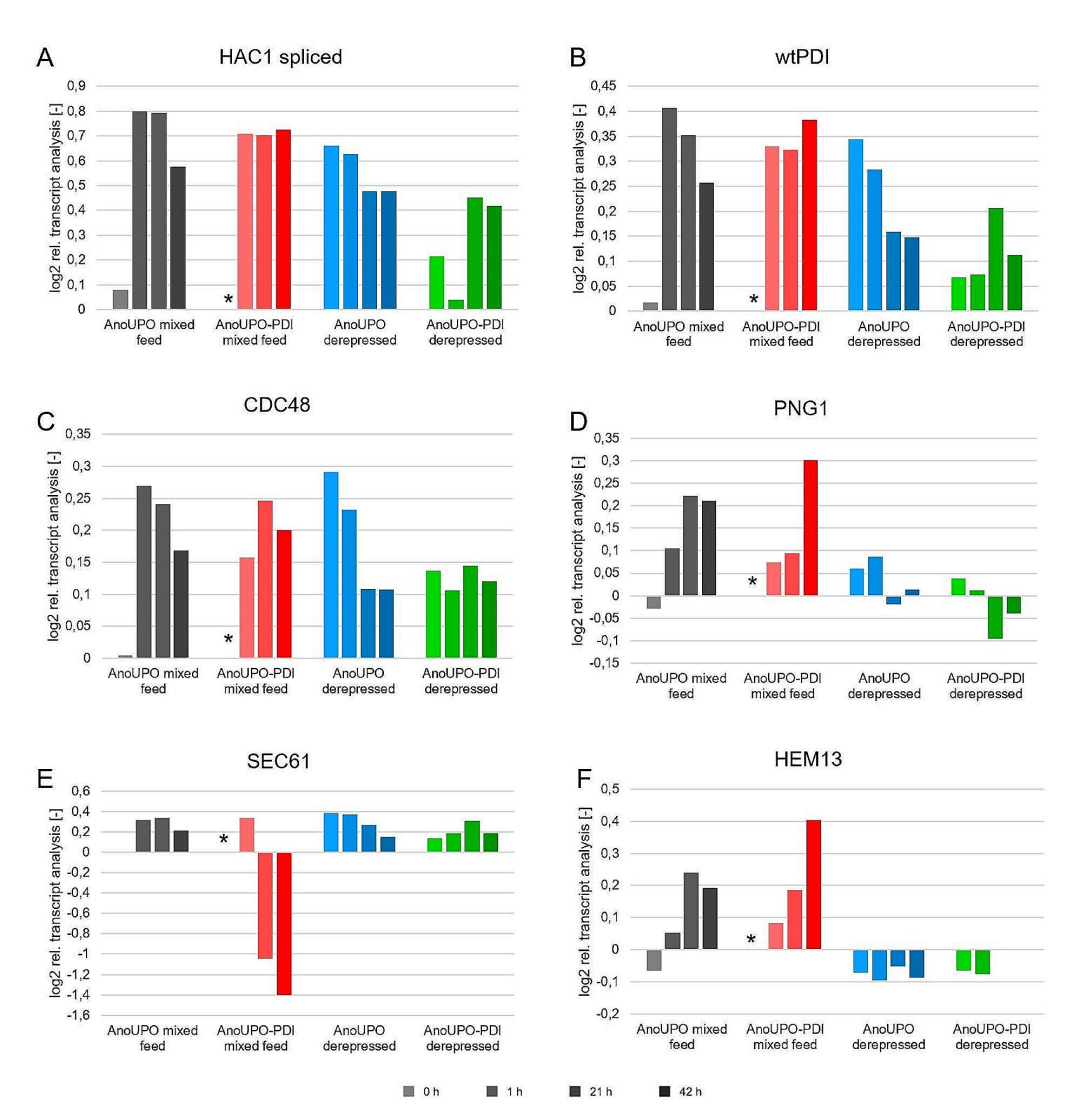
The two strains, *Ano*UPO and *Ano*UPO-PDI, were cultivated in a non-dynamic fed-batch mode and induced via mixed-feed or methanol-free derepression. Samples were taken at indicated induction time points: one sample prior to induction, a sample representing the switch proposed by the induction conditions (either switch in feeding rate or methanol pulse), and two further samples were taken in 20 h intervals to monitor the time effect of the induction period; the total RNA was isolated, and the relative transcript levels of the indicated genes were determined by an RT-qPCR assay normalized to the reference sample (indicated by an asterisk) using the reference genes *RSC1* and *TAF10* for normalization. Gene expression for these timed-dependent cultivations is shown for *HAC1* spliced (**A**), wt*PDI* (**B**), *CDC48* (**C**), *PNG1* (**D**), *SEC61* (**E**), and *HEM13* (**F**)

In contrast, we observed differences in the strength and timing of UPR induction among the various cultivations. The mediator of UPR, the spliced variant of *HAC1* as well as UPR targets, such as the wild-type *PDI* (wt*PDI*), *KAR2*, *CNE1*, and *Sect. 61*, were strongly upregulated in both the *Ano*UPO and the *AnoUPO*-PDI strain after the first methanol pulse and mostly stayed at these high levels until the end of the cultivation (Fig. 4A and B, additional data Sect. 1).

Literature suggests that the heme biosynthesis pathway is upregulated in *K. phaffii* when methanol is used to produce heme-containing recombinant proteins [19]. Hence, additionally to the target and UPR-associated genes, we analyzed transcript levels of *HEM12* and *HEM13*, encoding for the uroporphyrinogen decarboxylase and coproporphyrinogen III oxidase, respectively, the last two enzymes in the heme biosynthesis pathway [19]. While we could not observe an apparent and conclusive difference in *HEM12* transcription (addtional data Sect. 1), an elevated amount of *HEM13* transcript was observed in the mixed-feed cultivations for both strains compared to the derepressive cultures (Fig. 4F).

Also, the ATP-dependent protein import into the endoplasmic reticulum (= ER), catalyzed by the chaperone *KAR2* [67], was found to be upregulated in the methanol-induced compared to methanol-free cultivations. As *KAR2* is involved in the regulation of the unfolded protein response (UPR), its expression was additionally investigated.

Given that the co-expressed PDI is a foldase normally involved in the UPR (unfolded protein response), we measured the transcript levels of the native, as well as the co-expressed PDI and other UPR targets. In fact, we observed very high transcript levels for both the *Ano*UPO and the co-expressed PDI (co*PDI*) from the start of the induction phase, with a slight decrease over time, regardless of strain and cultivation method (additional data Sect. 1). In addition, the wt*PDI* was upregulated in methanol-induced mixed-feed cultivations compared to derepressed cultivations, indicating higher cellular stress (Fig. 3B). Both *HAC1* and wt*PDI* were upregulated in a reproducible manner when reproducing methanol induction during shake flask cultivation in triplicates.

Looking at the ratio of the *HAC1* spliced and *HAC1* un-spliced genes (Fig. 3A, additional data Sect. 1), it is clearly visible that upon the addition of methanol, the UPR significantly increased. This implies that host cells are subjected to a certain amount of cellular stress upon methanol addition. During derepressed feeding (Fig. 3A), the UPR was not affected immediately upon induction, suggesting that neither the *Ano*UPO expression nor the PDI expression per se were influencing UPR initially. The *HAC1* expression altered immediately upon the addition of methanol compared to pre-induction conditions. Still, over the time of induction, the UPR also increased in the derepressive cultivations, which presumably resulted from the higher cell-specific production of *Ano*UPO in the derepressed fed cultivation.

Additionally, *CNE1* and *SEC53*, two genes involved in the glycosylation transport chain to the ER, were also upregulated during methanol induction, indicating higher stress levels.

To shed further light on the differences in the selected process conditions, vesicular transport in the cultivations was analyzed using the genes *SEC61* and *SEC31*. *SEC61* is necessary for the import of secreted proteins into the ER lumen and is involved in the export of misfolded proteins from the ER lumen. *SEC31* is essential for the formation of ER-derived transport vesicles. Interestingly, the *Ano*UPO-PDI strain in the methanol-induced cultivation was found to show a decrease in transcripts for *SEC61* at later stages in the process (Fig. 3E). *SEC61* transcripts did not change significantly over the course of the derepressed cultivations. *SEC31* was also investigated, but no significant alterations of transcript expression could be detected throughout any cultivation (additional data Sect. 1). This might explain the breakdown in productivity of the process at later stages in the methanol-induced cultivations of both strains (Figs. 2B and 3B). Accordingly, derepressive feeding did not overstress the vesicle transport system (in regard to expression of *SEC61*), resulting in a presumably higher secretion of *Ano*UPO (Fig. 2B), while methanol induction seems to negatively affect the vesicle transport system (Fig. 4E).

Differences between both cultivation strategies were also observed in the ERAD (endoplasmic reticulum-associated protein degradation) pathway, indicated by the change in expression of various associated genes (*UBC1*, *CDC48*, *PNG1*, and *UBC7*) (Fig. 4C&D and additional data Sect. 1). Similarly to the UPR upregulation, we observed the strongest ERAD response in the *Ano*UPO strain after the methanol pulse and, on average, a milder ERAD upregulation in the *Ano*UPO strain in the derepressed cultivation (Fig. 4C and D, additional data Sect. 1). Notably, the different ERAD genes respond differently to the two different cultivation methods in both the *Ano*UPO and the *Ano*UPO-PDI strain (additional data Sect. 1). Here, the genes *UBC1*, *CDC48*, and *UBC7* showed higher expression levels during methanol feeding, indicating ERAD stress [68]. In addition, *PNG1* was highly upregulated in methanol-based inductions, indicating a higher requirement of the N-glycanase, being responsible for deglycosylating prior to degradation [69]. Higher N-glycanase activity thus indicates higher levels of protein misfolding in the methanol-induced cultivations.

Overall, the derepressive feeding exerted lower stress levels on the cells in direct comparison to methanol-based induction, and higher activities of the enzyme *Ano*UPO were found with derepressive feeding. Moreover, the chaperone PDI had a positive effect on the folding of *Ano*UPO, especially when high levels of the target enzyme were produced.

Notably, the transcript analyses were performed with single samples from the runs. To estimate if and how repeatable and thus reliable the obtained results were, we cultivated the *Ano*UPO strain and the *Ano*UPO-PDI strain in shake flasks in replicates and measured the transcript levels of the heterologously expressed genes (*Ano*UPO and co*PDI*) and the indicators for UPR (*HAC1* and wt*PDI*). We obtained results matching the production runs and the literature; this is, high expression levels of *Ano*UPO and co*PDI*, both boosted by methanol, elevated levels of the spliced *HAC1* transcript after the addition of methanol, and higher wt*PDI* expression in the *Ano*UPO strain compared to the *Ano*UPO-PDI strain (additional data Sect. 2). The determined standard deviation of the reproducibility experiments was below 4% on average.

To conclude, the RT-qPCR results indicated elevated cell stress during mixed feeding in comparison to derepressed feeding. Effects were clearly observed in the UPR-response (*HAC1* spliced-to unspliced ratio) as well as in the ERAD pathway (indicated by evaluated *PNG1, CDC48, UBC1*, and *UNBC7* levels over time). The RT-qPCR data revealed that PDI co-expression reduces UPR stress in the derepressed cultivation, as decreased *HAC1* and wt*PDI* levels were monitored throughout this experiment. Still, UPR levels were comparable for both strains during mixed-fed cultivations, indicating co-PDI expression to have no positive effect once methanol is added.

## Conclusion

For heme-containing unspecific peroxygenases, methanol-free bioreactor production strategies with *K. phaffii* are scarce in literature. To circumvent low productivities with methanol-free induction mechanisms, we evaluated if co-expression of the chaperone protein disulfide isomerase (PDI) could increase the titer and quality of a recently described short type UPO (*Ano*UPO). After screening the chosen clones in dynamic experiments, it could be shown that derepressive feeding of the bi-directionalized construct (*Ano*UPO-PDI strain) resulted in the highest enzymatic activity. Methanol induction was shown to stress host cells by upregulating UPR and the ERAD pathway, as well as lowering the protein secretion mechanism, compared to derepressed induction, determined by targeted RT-qPCR analysis. The co-expression of the chaperone PDI in a bi-directionalized manner favored the protein-specific activity, thereby bettering the enzyme quality and easing the purification of the desired target product.

Hence, in this study, we demonstrate a sustainable production strategy for UPOs, which has not yet been shown in literature according to the authors’ knowledge. We highlight that this promoter combination might be applicable for the production of diverse UPOs with *K. phaffii*, allowing further structure elucidation of this enzyme class.

### Electronic supplementary material

Below is the link to the electronic supplementary material.

Supplementary Material 1

## Data Availability

No datasets were generated or analysed during the current study.
