# Supplementary material for: Bi-directionalized promoter systems allow methanol-free production of hard-to-express peroxygenases with Komagataella Phaffii
Source: Microb Cell Fact. 2024 Jun 15;23:177. doi: 10.1186/s12934-024-02451-9 (PMC11179361; doi:10.1186/s12934-024-02451-9)
Supplement: Supplementary file 1 — Supplementary Material 1 [file 12934_2024_2451_MOESM1_ESM.docx]

**Bi-directionalized promotors systems allow methanol-free production of hard-to-express peroxygenases with *Komagataella phaffii***

Mihail Besleaga^1,a^, Christian Zimmermann ^1,b^, Katharina Ebner^2,c^, Robert Mach^1,d^, Astrid Mach-Aigner^1,e^, Martina Geier^2,f^, Anton Glieder ^2,g^, Oliver Spadiut^1,h^, Julian Kopp^1,i, §^

^1^ Institute of Chemical, Environmental and Bioscience Engineering, TU Wien, Gumpendorfer Strasse 1a, 1060 Wien, Austria

^2^ bisy GmbH, Wünschendorf 292, 8200 Hofstätten an der Raab

^a^ Email: mihail.besleaga@tuwien.ac.at

^b^ Email: christian.zimmermann@tuwien.ac.at

^c^ Email: katharina.ebner@bisy.at

^d^ Email: robert.mach@tuwien.ac.at

^e^ Email: astrid.mach-aigner@tuwien.ac.at

^f^ Email: martina.geier@bisy.at

^g^ Email: anton.glieder@bisy.at

^h^ Email: oliver.spadiut@tuwien.ac.at

^i^ Email: julian.kopp@tuwien.ac.at

§ Correspondence and Requests for materials should be addressed to:

Julian Kopp, TU Wien, Institute of Chemical, Environmental and Bioscience Engineering, Research Division Integrated Bioprocess Development, Gumpendorfer Straße 1a, 1060 Vienna, Austria. Tel.: +43 1 58801 166485, Email: julian.kopp@tuwien.ac.at

**Section 1: qPCR data bioreactor cultivations**

All qPCR data of bioreactor cultivations are given for the induction time-points:

- 0h
- 1h
- 21h
- 42h

**Expression of target enzyme *Ano*UPO via P*_DF_* promotors:**

**Expression of coPDI via P*_DC19_* promotors** (only expressed in the *Ano*UPO-PDI strain)**:**

**Unfolded Protein Response (=UPR) related genes**

1. HAC1: Activator of UPR (unspliced = inactive form)
2. HAC1: Activator of UPR (spliced = active form)
3. Other UPR targets (chaperones, quality control, etc.):

wtPDI: wildtype PDI = PDI expressed in wildtype strain

KAR2: ATP dependent protein transport into ER

CNE1: endoplasmic reticulum chaperone

SEC53: folding aiding for secretion of proteins from endoplasmic reticulum

SEC 61: protein import into ER and export of misfolded proteins

SEC31: essential for ER-derived transport vesicle formation

**ERAD (Endoplasmic-reticulum-associated protein degradation) targets:**

UBC1: mediates selective degradation of short-lived and abnormal proteins; plays a role in vesicle biogenesis and ER-associated protein degradation (ERAD)

CDC48: ATPase with protein-unfoldase activity; subunit of polyUb-selective segregase complex involved in ERAD

PNG1: deglycosylating enzyme that cleaves N-glycans that are attached to misfolded ERAD substrate glycoproteins

UBC7: Ubiquitin conjugating enzyme; involved in the ER-associated protein degradation (ERAD) pathway

HRD3/DER1: degradation of misfolded ER-lumen proteins

**Hemin biosynthesis:**

HEM12 codes for 5^th^ step in hemin biosynthesis

HEM13 codes for the 6^th^ step in hemin biosynthesis

**Section 2: qPCR data shake flask cultivations conducted in triplicates**

Cultivations were exercised in triplicates investigating the effects of starvation i.e. derepression (= Starv), methanol addition (MeOH) and growth on Glycerol in excess (Gly).

All cultivations were monitored for *Ano*UPO expression, coPDI expression, HAC1 (spliced and unspliced) as well as wtPDI expression after 8h of cultivation.

**Expression of target enzyme *Ano*UPO via P*_DF_* promotors:**

**Expression of coPDI via P*_DC19_* promotors** (only expressed in the *Ano*UPO-PDI strain)**:**

HAC1: Activator of UPR (unspliced = inactive form)

HAC1: Activator of UPR (spliced = active form)

wtPDI: wildtype PDI = PDI expressed in wildtype strain

**Section 3: Primer list for qPCR analysis**

| TAF10_fwd | CCTCACATTTCTATGCCTTCCC |
| --- | --- |
| TAF10_rev | GGCTTGAGGATTGGCACTAG |
| RSC1_fwd | CACGATGCCTGCTGATGTGG |
| RSC1_rev | GGGTACATACACTGGAGGACCTC |
| HEM13_fwd | CAATTCCATCAAGCCCATAAG |
| HEM13_rev | TCGAGTGATAGCGGGATAAGA |
| AnoUPO_fwd | GATAACCATGACTTCAACCAGG |
| AnoUPO_rev | ACAAGTATTCGACCCAGCTTC |
| coPDI1_fwd | GTTGGAAAGGCTCATGATGAG |
| coPDI1_rev | CGTCATACAGCTGTGGATTTG |
| wtPDI1_fwd | TCACTGTAAGAGAATGGCTCCTG |
| wtPDI1_rev | CGAGATCCATCATACAGTTGAGG |
| HAC1_fwd | GTCCGATGAGAACTTCTTGTTGAG |
| HAC1(unspliced)_rev | CTGTAATGTGTAAAGATGAATCCGTC |
| HAC1(spliced)_rev | GGTAAATGGTGCTGCTGGATG |
| KAR2_fwd | TATTCCACCAGCTCCAAGAGG |
| KAR2_rev | GAATGAGCGTAGTTCTCCAGAGC |
| ERO1_fwd | GTTGGAAAAGCCGCATATAAAC |
| ERO1_rev | AGCTTGGGCAAAGTCCTGTAAG |
| HRD3_fwd | TTATGATTTGGCTCTTCGAAGG |
| HRD3_rev | GTCATTTTGGTAAACATATCAGCG |
| DER1_fwd | TCAGGTTATTATTGGAAGGTCG |
| DER1_rev | CAAAGATTCGTCAGCCAGTTAG |
| SEC61_fwd | CTTCAAGTGACTCCTCTGACCC |
| SEC61_rev | GGAACAACTCTCTGTCTGCTTTG |
| PNG1_fwd | GTCCATCTGGATAGTTGCGAG |
| PNG1_rev | TGACCCGCTCTCTTTTCTATTC |
| SEC31_fwd | CACCAGCTATCCCTGTTGCC |
| SEC31_rev | GGTTGCACATGTTGAGCTAGTG |
| SEC53_fwd | CTCGGAAAACGGTTTGACTG |
| SEC53_rev | CTGATGTTGTGCTCCTTGTCG |
| CNE1_fwd | CTGAAACCCCACCTGAAACTG |
| CNE1_rev | GACCAAACGGCTACAAGGAAG |
| HEM12_fwd | TGCAGTTGAATTCTTAGCTCAGC |
| HEM12_rev | GGACAGCATCACTTGGACGG |
| UBC7_fwd | TTGACATTTCCTAAGGATTACCC |
| UBC7_rev | ATCTATGTTGGCACCGCTTTC |
| UBC1_fwd | ATGCCTGGACGCCTATTCTC |
| UBC1_rev | CTTCCGATTATTCTCATCCTGC |
| CDC48_fwd | CGGAGCATCTGATCGAGTTGT |
| CDC48_rev | GAGAGATCTGCTCCTGAAAACC |
